# A Design of Experiments Strategy to Enhance the Recovery of Polyphenolic Compounds from *Vitis vinifera* By-Products through Heat Reflux Extraction

**DOI:** 10.3390/biom9100529

**Published:** 2019-09-25

**Authors:** Mirela L. Moldovan, Sonia Iurian, Cristina Puscas, Radu Silaghi-Dumitrescu, Daniela Hanganu, Catalina Bogdan, Laurian Vlase, Ilioara Oniga, Daniela Benedec

**Affiliations:** 1 Department of Dermopharmacy and Cosmetics, Iuliu Hatieganu University of Medicine and Pharmacy, 12 I. Creangă Street, 400010 Cluj-Napoca, Romania, catalina.bogdan@umfcluj.ro (C.B.); 2 Department of Pharmaceutical Technology and Biopharmacy, Iuliu Hatieganu University of Medicine and Pharmacy, 41 Victor Babes Street, 400012 Cluj-Napoca, Romania; laurian.vlase@umfcluj.ro; 3 Department of Chemistry and Chemical Engineering, Babeş-Bolyai University, 400028 Cluj-Napoca, Romania, rsilaghi@chem.ubbcluj.ro (R.S.-D.); 4 Department of Pharmacognosy, Iuliu Hatieganu University of Medicine and Pharmacy, 12 I. Creangă Street, 400010 Cluj-Napoca, Romania, , dbenedec@umfcluj.ro (D.B.)

**Keywords:** *Vitis vinifera*, pomace, canes, polyphenolic compounds, antioxidant activity, design of experiments, waste management

## Abstract

The aim of the present study was to establish the best experimental conditions that lead to the extracts richest in polyphenolic compounds obtained from pomace and canes of *Vitis vinifera*. In this regard, a D-Optimal design of experiments (DoE) method was applied to investigate the extraction process parameters from each of three materials: red pomace (RP), white pomace (WP) and canes (C). The input variables were the extraction temperature and the ethanol ratio and as response, the total polyphenols content (TPC) was determined. A design space was generated for each of the plant materials and the most concentrated polyphenol extracts were obtained using 50% ethanol at a temperature of 80 °C. Further, the phenolic profiles of the concentrated extracts were detected by LC/MS/MS and the results showed that WP extract was richer in polyphenolic compounds, both flavonoid and phenolic acids, followed by the RP and C extracts. The antioxidant assays revealed that WP and RP extracts exhibited a higher antioxidant activity which correlated to the high content of polyphenols. These findings revealed that RP, WP and C, currently considered agricultural wastes from winery, may be valorized as an important source of natural antioxidants.

## 1. Introduction

The wine industry generates a high amount of by-products, the main residues being the pomace and the canes left from grape processing, that represent up to 30% (*w*/*w*) of the used plant material. The interest in valorization of these products is growing because they can be used as an easily available source of raw material for the recovery of a lot of bioactive substances for pharmaceutical and cosmetic applications instead of being treated as waste [1,2]. Of these bioactive materials, polyphenolic compounds are the most valuable due to their well-documented biological activities: antioxidant, antimicrobial, anti-inflammatory, cardioprotective, antiaging, and anticancer, hence the interest in their extraction [3,4,5,6,7].

One of the most notable bioactive properties is the antioxidant ability, including reduction of hydroperoxide formation, metal chelating properties, inhibition of lipid oxidation, and scavenging of free radicals [3,7]. The antioxidant activity reduces the oxidative stress, manifested when reactive oxygen species (ROS) are in excess. Even if ROS have important physiological roles, being involved in a lot of cellular processes including cell signaling and host defense system, their excess induces damages at cellular level. ROS superproduction is associated with the development of several chronic diseases such as cardiovascular disease, different types of cancer, Alzheimer disease, type 2 diabetes and osteoarthritis. Polyphenol administration has shown ameliorative or preventive effects in these pathologies, as reported in numerous studies [8]. In addition to that, their wound healing effect recommends them as actives in periodontal diseases. Thus, rosmarinic acid from *Salvia sclarea* L. decreased IL-1β, IL-6 and TNF-α levels and enhanced fibroblasts in gingival tissue [9]. The oligomeric proanthocyanidins from grape seed applied as a topical preparation on mandibular molar region bone lowered the inflammatory cell number and increased the connective tissue attachment level [10]. Moreover, the astringent character of tannins can contribute to the local contraction of the tissue and to a vasoconstrictor effect that reduces edema, exudation and inflammation [11]. For periodontal diseases, antioxidant and anti-inflammatory activities are very important, each of these being provided usually by chemical agents, whose side effects (e.g., limited period of use, disruption of oral microbiota and teeth staining) are well-known; therefore a mouth rinse containing a vegetal extract as active may represent an alternative, considering the need of long-term use in case of these oral diseases of a chronic nature.

According to some authors, a high significant positive correlation exists between the total phenolics and antioxidant capacity [12,13], which establishes as the main goal for any extraction process to get as much polyphenols as possible. A high antioxidant capacity is expected to lead to a high protective effect on human health.

In order to exploit the potential of by-products and to achieve high extraction efficiency, simple and robust extraction methods are needed. The mass-transfer and extraction efficiency can be modulated by varying the concentration gradient, diffusion coefficients and stationary layers, factors influenced by the type of solvent, plant material particle size, temperature, and duration of the extraction process. The critical parameters for the extraction process are the quality of plant sample, the solvent used for the extraction, the extraction procedure and the equipment used for extraction. Each factor may be described as an interaction of several subfactors as follows: the part of the plant, water content and degree of grinding of the herbal product; the type, ratio, concentration, volume and flow rate of the extraction solvent; the extraction procedure: type, duration, pressure and temperature and concerning the equipment used for extraction: the filling volume, the static pressure and the sample size [14].

Several extraction methods are mentioned for the recovery of polyphenols: conventional methods, based on solid-liquid extraction with different solvents, and novel methods, based on ultrasound or microwave assisted extraction, supercritical fluid extraction, hydrostatic pressure extraction or pulsed electric field systems. Among these methods, solid-liquid extraction remains one of the most commonly used extraction procedures due to its efficiency, ease of use and wideranging applicability [6]. Due to the complex biochemical composition, there is no universally accepted method to extract all antioxidant compounds. Thus, supplementary studies are needed for the extraction and recovery procedures in order to obtain standardized and well characterized extracts that can be used for further applications, such as cosmetic or pharmaceutical development.

The solvent choice for extraction is the most important variable in the extraction process, as the yield and the rate of polyphenolic extraction are related to solvent characteristics. Even if methanol has been reported as being more efficient for the extraction of low-molecular weight polyphenols and acetone for the high molecular weight polyphenols, ethanol-water mixture is often considered [6,14,15,16]. Its safety and environmentally friendly character recommends it for both pharmaceutical and cosmetic applications [17].

The Design of Experiments (DoE) approach is a helpful tool used in several industries to optimize products or technological processes. It is a scientific method based on statistical calculations that offers the optimal solution in a time-efficient manner, with the minimum number of experimental runs. Moreover, DoE facilitates the broad understanding of a process, of the connections between input and output variables and leads to high flexibility and predictability [18].

This manuscript describes the way in which DoE, a risk management tool within the QbD concept commonly used to investigate the major influences of formulation and process factors on critical quality attributes, was integrated into the strategy of winery industry by-products’ valorisation. The objective of this study was to increase the efficiency of the solid-liquid extraction of polyphenolic compounds from pomace and canes of *Vitis vinifera,* for the further use in mouth rinses as a part of the preventive and supportive strategy for the management of periodontal diseases. Thus, a D-Optimal optimization design was applied and the obtained results were used to generate the design space that reveals the combination of factors which ensures the maximum polyphenols content. Also, the antioxidant properties of the concentrated extracts were determined through five methods, in order to confirm their potential as a source of natural antioxidants.

## 2. Materials and Methods

### 2.1. Plant Material

Vegetable products consisting of dried grape pomace and canes of some varieties of *Vitis vinifera* were harvested from the experimental fields of SCDVV Murfatlar (44°10′25″N 28°24′30″E, Constanta County, Romania), in 2018. The sample RP is a mixture of equal parts of red pomace from varieties of *Vitis vinifera:* Pinot Noir, Feteasca neagra, Cabernet Sauvignon and Mamaia. The WP sample is the mixture of equal parts of white pomace from the varieties Muscat Ottonel and Sauvignon Blanc. The sample C was a mixture of equal parts of canes from all these varieties. The grape pomace was dried in a thin layer at ambient temperature in the deposit; it was aerated by palletizing to remove water and avoid the development of microorganisms each 24 h. The plant materials were reduced to a powder of a proper degree of fineness. In this sense, the vegetable products were ground in a coffee grinder (RC-21 Electroarges, Arges, Romania) for 5 min. Then, the powders were screened through a 200 µm sieve (Retsch, Haan, Germany) [19,20,21].

### 2.2. Chemicals

Gallic, ferulic, gentisic, acids, patuletin, luteolin were purchased from Roth (Karlsruhe, Germany), and caftaric acids were purchased from Dalton (Toronto, ON, Canada). Rutin, isoquercitrin, quercitrin, hyperoside, quercetin, and *p*-coumaric acid were acquired from Sigma (St. Louis, MO, USA). Folin-Ciocalteu reagent, sodium carbonate, sodium nitrite, sodium molybdate, sodium hydroxide, sodium carbonate, hydrochloric acid, aluminum chloride, sodium acetate, 2,2-diphenyl- 1-picrylhydrazyl (DPPH), 2,4,6-tris(2-pyridyl)-s-triazine (TPTZ), ferric chloride, Trolox (6-hydroxy- 2,5,7,8-tetramethylchroman-2-carboxylic acid), ethanol, HPLC grade methanol, acetic acid, analytical grade orthophosphoric acid, (+)-catechin, (−)-epicatechin, vanillic acid, syringic acid, protocatechuic acid (3,4-dihydroxybenzoic acid) were purchased from Sigma-Aldrich (Steinheim, Germany), Merck (Darmstadt, Germany) and Alfa-Aesar (Karlsruhe, Germany). Skin hide powder batch B6s was acquired from BLC Leather Technology Centre Limited (Northampton, UK). Soy lechitin was purchased from Sigma Aldrich. Hemoglobin was purified from bovine blood according to the protocol described previously by Antonini et al. [22]. Thus, the bovine blood, diluted in isotonic solution of NaCl 1% (1:5) was centrifuged 15 min at 2000 r.p.m. The red blood cells were diluted with cold ultrapure water (1:3) and washed with a solution of ammonium sulphate (4:1) and centrifuged at 1000× *g*, for 30 min. The precipitate was removed and the supernatant was dialyzed in 5 mM phosphate, pH 7.4 (36–48 h) [23,24,25,26].

### 2.3. Preparation of Grape Pomace Extracts (RP, WP) and Canes Extracts (C) from Vitis Vinifera, with High Polyphenolic Content/DoE for Extraction Efficiency Enhancement

#### 2.3.1. Experimental Design Development

The first objective of this study was to find the extraction method that leads to the richest extracts in polyphenolic compounds, obtained from pomace and canes of *Vitis vinifera*. Several extraction processes were carried out following a D-optimal experimental design developed using Modde software version 12.1 (Sartorius Stedim Data Analytics AB, Umea, Sweden). The experimental design included three sources of variation: the type of plant material (X1), the ratio of ethanol (X2) and the extraction temperature (X3) (Table 1). 18 different single experiments were generated plus 3 replicated runs used to calculate the degree of freedom and the reproducibility, thus a total of 21 extracts. The dependent variable or the response in the experimental design was the total polyphenolic content (Y1).

#### 2.3.2. Extract Preparation

As extraction methodology, the plant materials (RP, WP and C) were reduced to a proper degree of refinement (particle size ≤ 200 µm). The extraction was made by reflux method, on water bath, for 30 min at three different temperatures (50 °C, 65 °C and 80 °C) with aqueous ethanol solution in concentration of 40, 50, and 60% (*v*/*v*). The solid/solvent ratio was 1:10 (g/mL) [21,27,28]. The samples were then cooled down and were filtered through a paper filter in a 20 mL graduated flask, and the resulting residue was washed twice with ethanol in the same flask to a 20 mL volume. The samples were centrifuged (1930× *g*) for 20 min, and the supernatants were recovered. All assays were executed in triplicate.

#### 2.3.3. Determination of Total Phenolic Contents (Total Polyphenols, Tannins, Flavonoids, Caffeic Acid Derivatives)

The total polyphenol content (TPC) in the extracts was spectrophotometrically determined by using the Folin-Ciocalteu reagent. Gallic acid was used to set up the standard curve (R^2^ = 0.998), and the results were expressed as mg of gallic acid equivalents (GAE)/g dry material [29,30,31,32,33,34]. Standard calibration curve was made with gallic acid plotted at 0.02, 0.04, 0.06, 0.08 and 0.10 mg/mL and prepared in methanol: water (50:50, *v*/*v*). The TPC in the 21 samples (N1-N21) was determined by slightly modified Folin-Ciocalteau method: each ethanol extract (0.5 mL) was mixed with 1.0 mL Folin-Ciocalteu reagent, 10.0 mL distilled water and 290 g/L sodium carbonate solution in a 25 mL graduated flask. The samples were incubated in the dark for 30 min before measuring the absorbance at 760 nm by using water as compensation liquid. TPC values were determined using the equation and slopes generated from the calibration curve of gallic acid graph and were expressed as mg of GAE.

In order to quantify tannins by UV-spectrophotometry, the colorimetric Folin-Ciocalteu method was used [35,36]. The quantification of tannins by spectrophotometrically Folin-Ciocalteu method allows to estimate the total content including the polymers (hydrolyzable and condensed derivatives). The total tannins determination was indirectly performed by using the skin powder as a precipitating reagent and Folin-Ciocalteu reagent for color forming, respectively. The reaction product is the result of non-specific oxidation with polyphenols, the color is not only due to tannins, and complexation of tannins with skin powder is inevitable. First, the total polyphenolic compounds were determined by using Folin-Ciocalteu method. Secondly, the same extracts were treated with 100 mg of skin hide powder, and the tannin-protein insoluble complexes were removed by centrifugation for 20 min at 4500 rpm; the clear supernatant (non-adsorbed polyphenols solution) was treated with Folin-Ciocalteu reagent as mentioned above. Tannin content was calculated as a difference between total and non-tannin phenolic content [35,37,38,39,40].

The previously described aluminum chloride colorimetric method was used to quantify the total flavonoid content (TFC) in our samples. Five milliliters of each extract were mixed with 5.0 mL of sodium acetate 100 g/L, 3.0 mL of aluminum chloride 25 g/L, and filled up to 25 mL with methanol in a calibrated flask. The absorbance was measured at 430 nm. The total flavonoid content value, expressed as rutin equivalent (RE), was determined using a calibration curve based on rutin (R^2^ = 0.999). The flavonoid contents were expressed as mg rutin equivalents RE/g dry material [30,36].

The caffeic acid derivatives content was spectrophotometrically determined with Arnows’ reagent (10.0 g sodium nitrite and 10.0 g sodium molybdate made up to 100 mL with distilled water). The results were calculated by using an equation that was obtained from a calibration curve based on caffeic acid (R^2^ = 0.994) and were expressed as caffeic acid equivalent on dry plant material (mg CAE/g) [30,36].

The equipment used for all the absorbance measurements was an Agilent Cary 60 UV-VIS spectrophotometer (version 6860A, SN: MY18370014, Agilent, Santa Clara, CA, USA) equipped with the Cary UV-Win software (5.1.1.-P/N: G6861-64001), a cell holder, and 1 cm matched quartz cells.

#### 2.3.4. Experimental Design Analysis and Extraction Efficiency Enhancement

TPC values were included as responses into the experimental design software Modde version 12.1 and analyzed for data fitting and statistical parameters calculation. The model significance was evaluated by ANOVA test (*p* < 0.05). The effects of independent variables were assessed by using Multiple Linear Regression method. The surface response plots were generated to illustrate the effect of each input variable on the TPC. Further, the software was used to generate the conditions to obtain the highest TPC in the form of a design space for each plant material.

### 2.4. LC/MS Analysis of Polyphenols

In the present study, the polyphenolic profile of our extracts was analysed by using liquid chromatography–tandem mass spectrometry (LC/MS), according to a previously validated and described method [31,32,41]. The experiment was performed on an Agilent 1100 HPLC Series system (Agilent, Santa Clara, CA, USA) equipped with G1322A degasser, G13311A binary gradient pump, column thermostat, G1313A autosampler, and G1316A UV detector. The HPLC system was coupled with an Agilent Ion Trap 1100 SL mass spectrometer (LC/MSD Ion Trap VL).

LC-MS analysis of the RP, WP and C extracts was performed according to a previously described method and slightly modified (replacing of sodium phosphate with acetic acid in the mobile phase) [31,32,41]. In brief, separation of the compounds was carried out on a Zorbax SB-C18 reverse-phase analytical column (100 mm × 3.0 mm i.d., 3.5 μm particle) with a mixture of methanol: 0.1% acetic acid (*v*/*v*) as mobile phase and the flow rate was constant at 1 mL/min and a linear gradient was maintained at 5% methanol and 42% methanol over the first 35 min, followed by isocratic elution at 42% methanol for 3 min. Oven temperature was maintained at 48 °C while detection was achieved at 330 nm and 370 nm by using a G1311A diode array detector system. Injection volume was 5 μL and data were collected at 330 nm wavelength. The detection of the compounds was performed on both UV and MS mode. The UV detector was set at 330 nm until 17 min (for the detection of polyphenolic acids, then at 370 nm until 38 min to detect flavonoids and their aglycones. The mass spectrometer was equipped with turboion spray (ESI, electrospray ionization) interface, negative ion mode. The identification of the polyphenols in the samples was made by comparing the retention times with those of the pure standards of phenolic compounds. Quantification was based on linear calibration plots of the peak area against concentration. Dilutions of the phenolic standard solutions were made at five calibration level between the ranges of 0.5 and 50 mg/L. All compounds were identified by comparison of retention times and the MS spectra with those of the standards, using same parameters and chromatographic conditions. The data were processed by ChemStation and Data Analysis software from Agilent.

In order to identify polyphenols such as epicatechin, catechin, siringic, gallic, protocatechuic and vanilic acids, another HPLC-MS method previously described by other authors was used [21]. Briefly, working conditions: Zorbax SB-C18 column, mobile phase of methanol:0.1% acetic acid (*v*/*v*) and a binary gradient (start: 3% methanol, at 3 min: 8%:20% methanol; keep 20% methanol until 10 min then rebalance column with 3% methanol); flow rate was 1 mL/min and the injection volume was 5 μL. The detection was performed on MS mode. The MS system operates by using an electrospray ion source in negative mode (capillary +3000 V, nebulizer 60 psi, dry gas nitrogen at 12 L/min., dry gas temperature 360 °C). The polyphenol compounds were determined on their peak areas and compared to a calibration curve of their six corresponding standards (epicatechin, catechin, siringic, gallic, protocatechic and vanilic acids) [21]. The results were expressed as micrograms of phenolic per gram of dry plant material.

### 2.5. Determination of Antioxidant Properties

The antioxidant activity of plant extracts was determined by several in vitro methods: the DPPH bleaching, ferric reducing powers (FRAP), nitrite-induced hemoglobin autoxidation (NHA), inhibition of lipid peroxidation catalyzed by cytochrome *c*, and free radical generation experiment.

#### 2.5.1. DPPH Assay

The DPPH test is a widely used method due to the short time required for analysis and is based on the scavenging of DPPH radical in the presence of hydrogen donating antioxidants (e.g., polyphenols) due to the formation of non-radical form DPPH-H. The free radical scavenging activity of the pomace and canes extracts and Trolox as a standard reference compound was analyzed by using DPPH assay as described earlier with some modifications [19,20,42,43,44]. In this assay, briefly, 30 μL of each extract of different concentrations (37.5–262.50 μg/mL) was mixed with 2 mL of methanolic DPPH solution (0.1 g/L). After 30 min. of incubation at 40 °C in a thermostatic bath, the decrease in absorbance was measured at 517 nm. The percent of DPPH scavenging ability was calculated from the absorption according to the following equation I%: = (A_control_ − A_sample_/A_control_) × 100, where A_control_ is the absorbance of DPPH radical and methanol (containing all reagents except the extracts) and A_sample_ is the absorbance of DPPH radical and extract mixture. The scavenging effect on the DPPH radical of the samples was calculated as the Trolox equivalent’s antioxidant capacity from the calibration curve: y = 2.848 x + 18.08, R^2^ = 0.997, obtained by Trolox standard solutions (5–25 µg/mL). The results were also defined as inhibitory concentration IC_50_.

#### 2.5.2. Ferric Reducing Antioxidant Power (FRAP) Assay

The antioxidant capacity of the extracts was estimated by the spectrophotometrically ferric reducing/antioxidant power (FRAP) assay following the procedure of Benzie and Strain [20,43,45]. The FRAP method relies on the change in the color of a complex with Fe^+3^ ion of the TPTZ radical (colorless complex) by the reduction of the ferric ion to Fe^+2^-tripyridyltriazine (a blue colored complex) formed by the action of electron donating antioxidants at low pH. To 2.5 mL of 10 mM TPTZ solution in 40 mM HCl, 2.5 mL 20 mM ferric chloride solution and 25 mL acetate buffer (pH = 3.6) were added. This mixture is the FRAP reagent. 6 mL the FRAP reagent were added to 0.4 mL of diluted sample and the absorbance value was measured. A blank solution was prepared in the same manner by using water instead of sample. Trolox was used as a reference. The color change was correlated with the antioxidant capacity by measuring absorbance at 450 nm. Using a calibration curve (R^2^ = 0.992) constructed with 10–40 mg/L Trolox standard, the results were converted to µmoles of Trolox equivalents/100 mL extract.

#### 2.5.3. Nitrite-Induced Autoxidation of Hemoglobinin

The reaction of hemoglobin with nitrite was studied at 540 nm, in phosphate buffer 50 mM, at pH 7, at room temperature. 40 µM oxy-hemoglobin were mixed with 166 µM nitrite in the presence of extracts. The inflection time (t_i_) was calculated by using Origin Pro 8 (OriginLab Corporation, Northampton, MA, USA). The results are given in mg CATE (catechin)/g plant using a calibration curve where R^2^ = 0.98 [23]. This project was approved by the Commission for Bioethics and Research Ethics of the Veterinary Sanitary and Food Safety Authority of Cluj-Napoca (approval No. 73/14.06.2017). 2.5.4. Inhibition of lipid peroxidation catalyzed by cytochrome *c*.

Liposomes were obtained by sonication of 5 mg/mL soybean lecithin, in phosphate buffer 10 mM, at pH 7. The reaction was catalyzed by 2 µM cytochrome *c* in the presence of extracts (16.7 µg/mL) and was monitored in time at 235 nm for 14 h [24].

#### 2.5.4. Free radical Generation Experiment

For the EPR experiment the extracts were diluted at 0.5% in 90% ethanol, then 5 mM NaOH were added to the solution according to the protocol described in [26]. The EPR spectra were recorded in a capillary glass EPR tubes from Hirschmann (Eberstadt, Germany), at room temperature with an ELEXSYS E-580 spectrometer (Bruker, Billerica, MA, USA) with continuous wave at X band (~9.4 GHz, modulation amplitude, 1 G, microwave power, 9.6 mM, center field 3514, sweep field 100 G, number of point 2014, conversion time 10 msec).

### 2.6. Statistical Analysis

The samples were analyzed in triplicate or more; the average and the relative were calculated by using the Microsoft Excel 2016 (Microsoft Corporation, Redmond, WA, USA) software package. The Gaussian distribution was checked by the Shapiro-Wilk normality test. Statistical analyses were performed by two-way ANOVA followed by Bonferroni post-test. Statistical significance was at *p* < 0.05 (95% confidence interval). Statistical values and figures were obtained by using GraphPad Prism version 5.0 for Windows (GraphPad Software, La Jolla, CA, USA).

## 3. Results and Discussion

### 3.1. DoE for Extraction Efficiency Enhancement

#### 3.1.1. Fitting the Experimental Results with the Model

The experimental part presented in this paper is a preliminary study that tries to give solutions on how to reuse winery by-products. As it was estimated that over 70% of the polyphenolic content is left in the pomace after wine production, active ingredient extraction for pharmaceutical or cosmetic use is a promising option [5]. The purpose of the first phase of the study was the extraction process efficiency enhancement through DoE. DoE methodology was preferred to the empirical approach for providing a deeper understanding of the extraction process, of the interactions between variables and for the ability to propose optimal formulations within the experimental domain [46]. Table 2 shows the independent and dependent variables included in the experimental design. X_1_, a qualitative variable, encodes the type of plant material received from the winery industry, used for the extractions: red pomace (RP), white pomace (WP) and canes (C). Several literature reports highlight the impact of the extraction solvent on the process efficiency [12,47,48]. For safety and lack of toxicity reasons and due to preliminary experimental results on extraction efficiency (results not shown), an ethanol:water mixture was chosen as extraction solvent. Therefore, the first quantitative factor (X_2_) was the ethanol ratio which varied between 40% and 60%. Reports focusing on how temperature impacts polyphenol recovery are scarce, however studies using different extraction methods were performed at temperatures starting from 10 °C up to 110 °C [12,49,50]. For this study the temperature (X_3_) was varied in the range of 50–80 °C. The DoE software was used to generate an experimental design matrix from the aforementioned independent variables, shown in Table 2.

The 21 extracts were prepared according to the experimental design specifications. The resulting extract was meant to be used as an active ingredient in local preparations (such as mouth rinses) for the oral hygiene of patients with periodontal disease. Therefore, the most important quality criteria was the presence of antioxidant compounds which, as literature states, exert important anti-inflammatory, antibacterial and immunomodulatory effects [8]. Also, the presence of tannins in the extract would be an advantage as they grant an astringent effect to the preparation. TPC determination using Folin-Ciocalteu reagent was contested by many researchers because of its lack of specificity, as many other compounds like thiol derivatives, vitamin derivatives, amino-acids and carbohydrates show significant reactivity which can lead to a polyphenol content overestimation [51]. However, Everette et al. state that in most plants, phenolics are the most abundant antioxidants. Since many studies have shown the strong correlation between TPC and the antioxidant activity, this parameter was chosen as the dependent variable; it was assessed for all 21 extracts and listed in Table 2 [52,53]. Applying the design of experiments methodology results in an equation, called model, that describes the dependence between the response (Y), TPC in this particular case, and the input variables (X_1–3_):

Y = b_0_ + b_1×1_ + b_2_X_2_ + b_3_X_3_ + b_4_X_1_^2^ + b_5_X_2_^2^ + b_6_X_3_^2^ + b_7_X_1_X_2_ + b_8_X_2_X_3_ + b_9_X_1_X_3_ + b_10_X_1_X_2_X_3_ (1)

where Y is the dependent variable (response), b_0_ is the mean of the response values, b_1_-b_10_ are regression coefficients, X_1_, X_2_, X_3_ are individual effects which show how the response varies due to one factor when all the others are kept constant. X_1_^2^, X_2_^2^, X_3_^2^ indicate non-linear dependence on the corresponding factor. X_1_X_2_, X_2_X_3_, X_1_X_3_ and X_1_X_2_X_3_ are interactive effects obtained when two or three factors change simultaneously [54].

**Figure 1:**
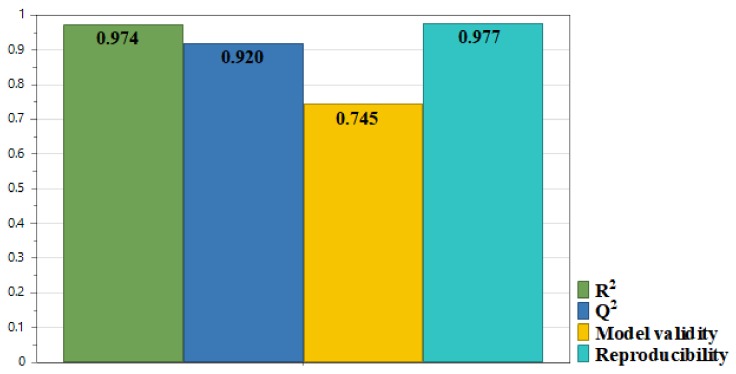
Summary of fit for the model corresponding to response Y, TPC.

The model coefficients were scaled and refined and the data showed a very good fit to the model (Figure 1). R^2^ indicates the response percentage explained by the model. It is a measure of fit, of how well the model fits the data. Its value is very close to 1; therefore it shows a high quality of fit. Q^2^ indicates the response variation percentage predicted by the model according to cross validation. It shows how well the model predicts new data. A high Q^2^ value corresponds to good prediction capacity models. Also, the difference between R^2^ and Q^2^ should be low, which in our case is less than 0.1. Model validity bar is higher than 0.25, which shows no lack of fit, meaning that the model error is in the same range as the pure error. The reproducibility shows the variation of the response obtained under the same experimental conditions, compared to the total variation of responses. In our case, it is close to 1, which means that the pure error is close to 0 and the values obtained by working in identical conditions are almost the same.

#### 3.1.2. The Effect of Input Variables (X_1–3_) on TPC (Y)

TPC varied within the 21 formulations on a wide range, from 10.07 to 31.35 mg GAE/g of dry plant material which indicates that the independent variables were well selected. The coefficient plot shows the connection between the input variables and the response, TPC. The positive values (sign +) indicate a positive influence on the response, while the negative values show a negative influence on the response (sign −).

**Figure 2:**
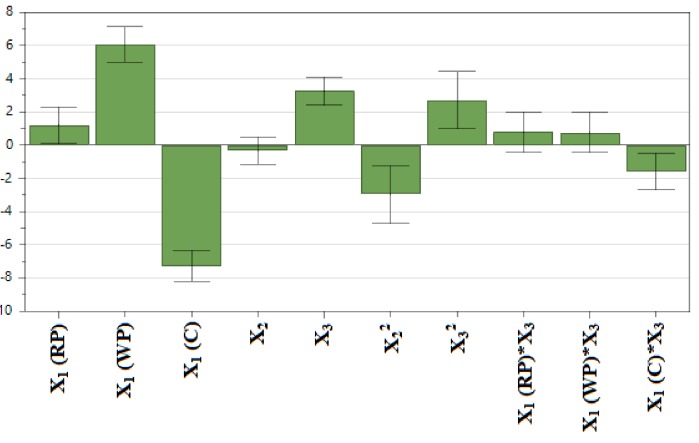
The factors influencing TPC. X_1_: plant material, RP: red pomace, WP: white pomace, C: canes, X_2_: ethanol ratio, X_3_: temperature.

It appears that the most important influence on total polyphenolic compounds was exerted by the type of herbal product: WP yielded the highest TPC, followed by the RP and finally by C (Figure 2). Pomace use had a positive influence on polyphenol extraction, meaning it led to higher TPC when compared to the canes, which had a negative influence on the extraction yield and resulted in low TPC values. The ethanol concentration had an insignificant effect (*p* > 0.05), but the quadratic term seemed to be significant, with a negative effect on the TPC, which shows a nonlinear dependence between the factor and the response.

**Figure 3:**
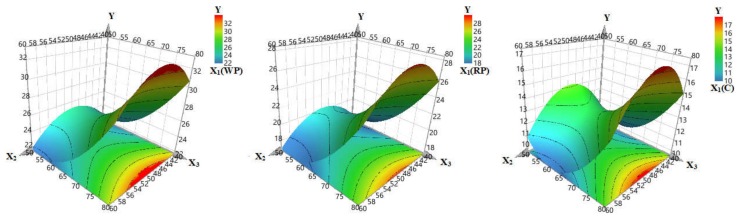
Response surfaces describing the total polyphenols content (Y) as a function of ethanol ratio (X_2_) and temperature (X_3_), for the three types of plant material (X_1_): white pomace (WP), red pomace (RP) and canes (C).

The temperature had a positive effect on the response, as well as the quadratic coefficient, which shows a significant dependence of the TPC on the temperature. High temperatures determine the extraction of large amounts of polyphenols. The nonlinear effects are better described in the response surfaces (Figure 3), where the TPC for each herbal product (WP, RP and C) was plotted against the temperature and the ethanol content.

For WP, which showed the best results (31.35 mg GAE/g dry material), the temperature had a positive effect on the response which increased abruptly at temperatures beyond 65 °C. The ethanol concentration increase up to 50% led to the increase of TPC, but further ethanol ratio increase led to lower TPC. The highest TPC obtained from RP was of 25.175 mg GAE/g plant material and the same variation was observed. Intermediate ethanol concentrations and high temperatures favored the extraction of polyphenols. With regard to canes, the largest amount of polyphenols was 17,375 mg GAE/g. The quadratic effects were more intense for both temperature and ethanol concentration. The curvature indicates that increasing temperature from 50 to 60 °C leads to a slight decrease in extraction yield, but further heating gives a significant TPC increase. However, the conclusion is the same, that intermediate ethanol concentrations coupled with high temperatures give the best extraction outcome. Our results confirm those reported in previous studies, that high extraction temperatures lead to higher polyphenols recovery, however the study domain was limited to 80 °C due to the oxidation degradation reactions that can affect the polyphenols’ integrity [49]. Also, higher temperatures could have promoted high solvent loss and low repeatability [49]. Regarding the extraction solvent, previous literature reports sustained the efficiency of polar solvents for phenolics extraction, with focus on the binary solvent-systems that seemed to give superior results to mono-solvent systems [21]. The ethanol ratio in the ethanol-water mixture had an important influence on the TPC, which showed good agreement with literature results. TPC values varied between 10.07 and 31.35 mg GAE/g, slightly lower than those obtained by Caldas et al. (2018) from the extraction of red pomace, which were comprised between 5.7 and 48.6 mg GAE/g [55]. However our results were significantly higher than those reported by Gonzales-Centeno et al. who performed power ultrasound assisted extraction in aqueous media and obtained a maximum of 770.9 mg GAE/100 g dry plant material [56].

#### 3.1.3. Extraction Method Efficiency Enhancement

**Figure 4:**
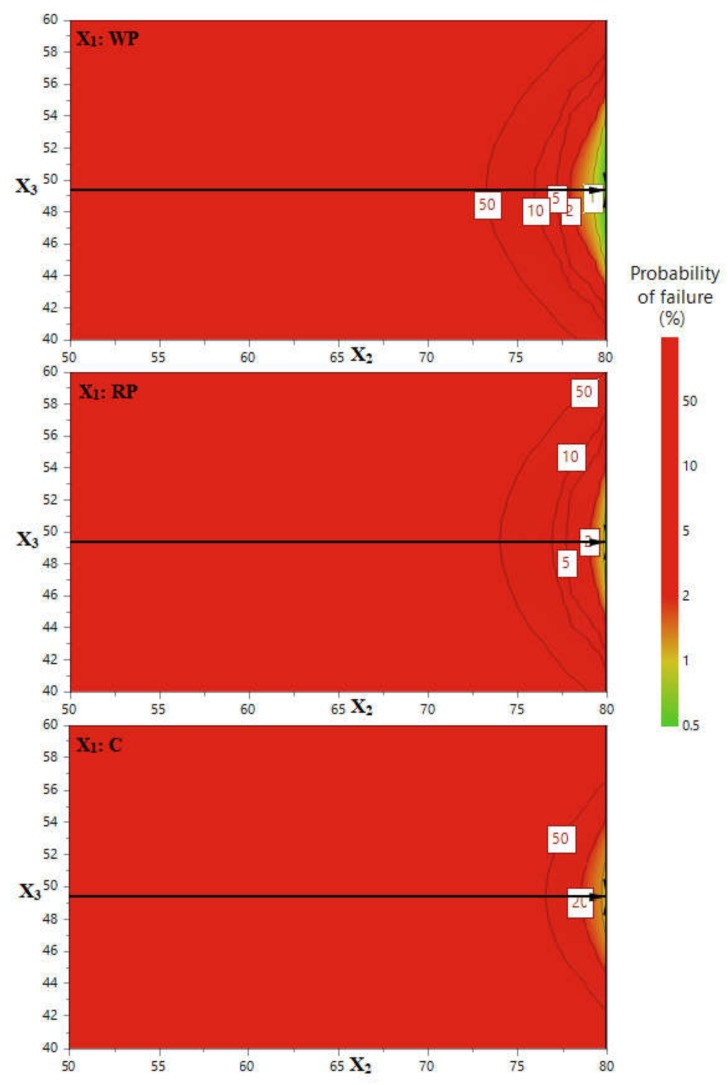
Design spaces describing the conditions necessary to obtain maximum extraction efficiency for the three types of plant material (X_1_): white pomace (WP), red pomace (RP) and canes (C), with X_2,_ the ethanol ratio (%) and X_3,_ the temperature (°C).

The objective of the first stage of the study was to optimize the polyphenols extraction for all the three plant products (WP, RP and C), to find the conditions needed for the highest extraction efficiency. The Modde software offers the means to define within the experimental region an area where these preconditions are fulfilled. It is called design space and it is defined as the multidimensional combination and interaction of input variables that were confirmed to assure the quality of the product [54,57]. For this particular study, the design spaces are composed of multiple interactions between the extraction temperature and the ethanol ratio, as a function of the qualitative variable. Therefore, three design spaces and thus three robust points were generated, corresponding to each of the plant materials, by applying one limitation in the experimental design software: the maximization of TPC. The design spaces are presented in Figure 4 together with the robust point indicated by the tip of the black arrow. For all the three plant materials the best extraction conditions were determined, with a certain probability of failure. Each point in the yellow area of the experimental domain corresponds to a temperature and to a specific ethanol content that grant 99% probability to get the highest TPC.

Apparently the best extraction conditions for all the three plant materials were 49.33% ethanol and a temperature of 80 °C. The medium ethanol content gave the best TPC results, which could be related to the different polarities that polyphenolic compounds in grape by-products have [55]. These results are in agreement with the work of Caldas et al. who studied the influence of the solid: solvent ratio, as well as of ethanol ratio and obtained the highest efficiency for a solid: solvent ratio of 1:10 and 50% ethanol [55].

### 3.2. Extract Characterization

The TPC were determined for all 21 samples in different conditions (Table 2). The richest in polyphenols were the extracts N3 for RP (25.17 mg GAE/g), N9 for WP (31.35 mg GAE/g) and N18 for C (17.37 mg GAE/g), all obtained at 80 °C. The extraction conditions for the highest polyphenol concentrations were established as follows: 50% ethanol and 80 °C. The raw materials were extracted in these conditions and the TPC, total flavonoid content (TFC), caffeic acid derivatives and tannins were determined for these extracts using spectrophotometric methods (Table 3). TPC and tannins content were expressed in mg/g dry plant material as gallic acid equivalent (GAE)/g dry plant material. The total flavonoid content (TFC) was expressed as mg rutin equivalent (RE)/g dry plant material and caffeic acid derivatives as mg of caffeic acid equivalents (CAE)/g dry plant material. All the experiments were performed in triplicate.

The WP extract contained the highest amount of total polyphenols, flavonoids and phenylpropane derivates (37.80, 1.89, and 17.64 mg/g, respectively), followed by the RP extract (32.00, 0.54, and 9.11 mg/g, respectively). The lowest concentrations in these active principles were measured in grape canes extract (18.45, 0.33 and 1.86 mg/g, respectively), our results being comparable to those found by other authors [58,59,60]. Generally, the 50% ethanol extract of WP was more concentrated in TPC than those from 50% ethanol extract red pomace and canes. Similar trends were also observed for flavonoid and caffeic acid derivatives. Regarding the tannin content, all samples showed high concentrations, but the richest was the RP (18.26 mg/g). Similar results were obtained by other authors for the red and white grape pomaces [59]. Comparing our results with other authors’ results [12,50,51]) is difficult, especially because the raw materials are mixtures of different grape pomaces and canes from *Vitis vinifera* varieties grown in Romania. It can be concluded that the RP extract was the richest in tannins, while in the WP extract, the flavonoids and caffeic acids derivatives were more concentrated.

#### 3.2.1. Chromatographic Analysis of Phenolic Compounds

The investigation of phenolic profiles with LC/MS/MS in negative ion mode resulted in the tentative identification of 15 compounds (Table 4). The phenolic compounds were identified by comparison of their retention times and the MS spectra with those of the standards, using the same parameters and chromatographic conditions. The two types of grape pomace did not show identical phenolic profiles, flavonoids were more common in WP than in RP. Phenolic acids (gallic, protocatechuic, caftaric, syringic acids) were found in all samples. Gallic and syringic acids were determined in the largest quantities in RP (146.87 and 72.22 µg/g, respectively). Protocatechuic acid was almost 1.6 times more abundant in WP (57.17 µg/g) than in RP (34.88 µg/g) and about five times less in canes (11.69 µg/g) than in WP. Vanillic and *p*-coumaric acids were present only in the RP sample. With regard to catechin and its isomer, epicatechin, large amounts were obtained in all extracts (> 500 μg/g in WP, 425–561 μg/g in RP and less in C, 278.90 μg/g). Our findings seem to be supported by several previous studies [20,61,62]. Four flavonoid glycosides: hyperoside, isoquercitrin, rutin and quercitrine and two flavonoid aglycons, quercetin and luteolin, were found in our extracts, and the the WP extract was the richest in these components. Thus, isoquercitrin, quercitrine and luteolin were determined in much higher quantities in WP (29.70; 26.0; 3.50 µg/g respectively), than in other samples. Other compounds, such as caftaric, gentisic, *p*-coumaric acids and hyperoside were also identified in the extracts, but they were in too low concentration to be quantified. It can be concluded that grape by-products can be a rich source of antioxidant polyphenols such as catechin, epicatechin and gallic acid, especially WP which can have important potential for pharmaceutical uses.

#### 3.2.2. Antioxidant Activity

The evaluation of antioxidant activity of the extracts by DDPH method lead to IC_50_ values that increased in the order: WP < RP < C. IC_50_ values were calculated in the same concentration range 37.5–262.50 μg/mL. A lower IC_50_ value shows a good antioxidant capacity. The results of DPPH scavenging activity showed that the white grape pomace extract are potentially more active (76.33 μg/mL), than the red grape pomace extract (112.02 μg/mL), as shown in Table 5. Similar results were obtained for the Bolognese red and white grape pomace [61]. Regarding the antioxidant activity of red pomace, similar results have been obtained with a mixture of pomaces from the Alijó Cooperative Winery (Vila Real, Portugal) [63]. The extract from the canes of *Vitis vinifera* indicated a low antioxidant activity through this test (198.27 μg/mL and I% = 30.86, respectively). I% values around 30 were also obtained from canes harvested from different varieties of *Vitis vinifera* from Czech vineyards [58,64].

The reduction power of all ethanol extracts on ferric ion is also shown in Table 5. FRAP values ranged from 997.81 to 3908.42 µmol TE/g among the three samples. As in the DPPH test, the order was as follows: WP > RP > C, the grape white pomace extract exhibited a higher activity than red pomace and canes extracts Generally, this is in line with the observations made by other authors regarding antioxidant power FRAP of the pomace extracts, which reported values close to ours [44,64]. Instead, other Romanian authors have obtained higher values, over 500 µmol TE/g [65].

Using the more physiologically/biologically-relevant assay method involving the inhibition of nitrite-induced hemoglobin autooxidation (NHA), antioxidant activity order was relatively different from the DPPH case. In the NHA assay, nitrite induces the autooxidation of hemoglobin according to the previously described mechanism [23] in which the following types of processes occur: nitrite reacts with the ferrous-oxy form of hemoglobin to generate a ferric-peroxynitrate complex, which then decays to a ferryl (Fe(IV-oxo) hemoglobin; ferryl hemoglobin is further reduced to Fe(III) (met) by the excess nitrite—yielding NO_2_ radicals which then engage in chain reactions that also involve the formation of nitric oxide (NO) and other nitrosating agents—but can also interact with the remaining oxy hemoglobin molecules thereby accelerating their autooxidation and thus generating superoxide and subsequently hydrogen peroxide and hydroxyl radicals [23]. The antioxidants can inhibit these processes both by reacting with free radicals/oxidants in solution (which includes oxidative as well as nitrosative stress agents) and by reacting with high-valent protein-bound iron (Fe(IV)). In this experiment all the grapevine extracts were found to inhibit nitrite-induced hemoglobin autooxidation, but the best results were obtained for WP and RP, with no statistically significant difference between them (Table 5). This (and also the slightly higher average value for RP vs. WP) is in contrast to the DPPH data where WP displayed significantly higher reactivity than RP; this can be explained by the more complex nature of the NHA assay (both the protein-based character and the involvement of nistrosative stress agents), as well as by antioxidants other than polyphenols present in RP.

**Figure 5:**
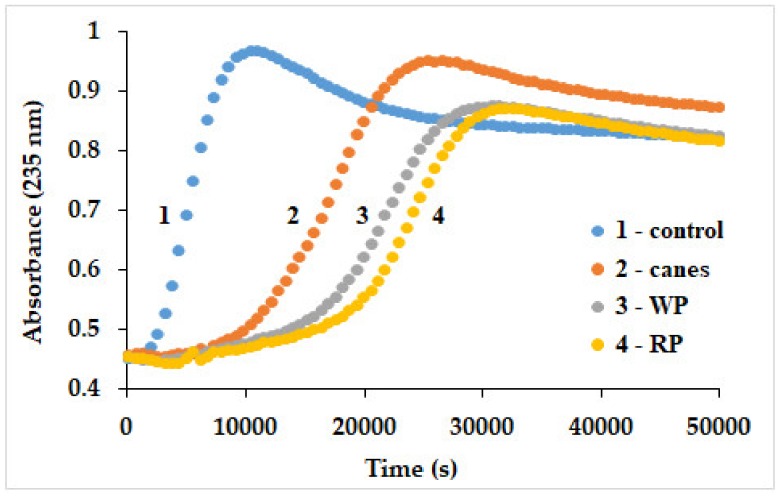
Time course for the oxidation of liposomes catalyzed by cytochrome *c* in the presence of the pomace (white and red pomace—WP, RP) and canes (C) extracts. Conditions: cytochrome *c*—2 µM, liposomes—0.5 g/mL, extracts—16.7 µg/mL, phosphate buffer—10 mM, pH 7.

Last but not least, in a protein- and lipid-based assay antioxidant assay (using a physiological reaction, the oxidation of liposome lipids catalyzed by cytochrome *c* monitoring conjugated diene formation spectrophotometrically [24,30,66]; the longest lag phase is correlated with the best antioxidant capacity), the order of reactivity was similar to the NHA assay, according to Figure 5: RP > WP >> C.

#### 3.2.3. Free Radicals Assessment

**Figure 6:**
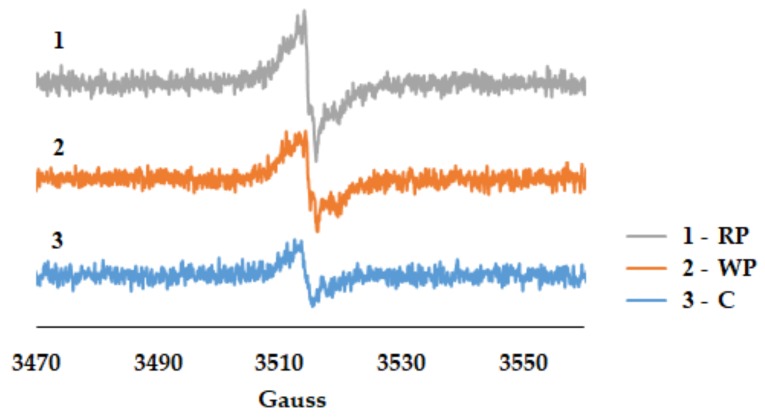
EPR spectra of the grape pomace (WP, RP) and canes (C) extracts treated with NaOH. Conditions: extracts–0.5%, NaOH—5 mmoles, Et-OH—90%.

Electron paramagnetic resonance (EPR) signals for pomace and cane extracts from *Vitis vinifera* are reported here in Figure 6 for the first time, using a protocol previously described for natural extracts and antioxidants (alkaline medium, to enhance autooxidation and hence longer-lived radicals generated mainly by polyphenolic species [26]; Notably, while EPR has previously been employed for analyzing such samples [67], it has always been restricted not to detecting signals generated by the extract itself, but rather signals of exogenous/artificial molecules such as DPPH or ABTS—and more exactly the decay of such signals due to the antioxidant reactivity of the extract; thus, this is the first report of the EPR signals of antioxidants from *V. vinifera* themselves. In this assay the intensity of the signal is generally correlated with antioxidant reactivity); these signals were weaker and less defined compared with signals detected by this same method in the natural extracts previously described—which precludes fingerprinting of the extracts by comparison with known signals of individual components (e.g., gallate, rutin, etc) [25,68]. Nevertheless, it can be observed that RP had the highest signal, followed very closely by WP. This result suggests that the highest content in antioxidants able to form in basic medium semiquinone radicals detectable by EPR belongs to RP, followed by WP and canes. These results are in agreement with the NHA and the liposome experiments.

## 4. Conclusions

Our study aimed to optimize the phenolic compounds extraction parameters from two grapes by-products (pomace and canes) as well as to evaluate the content of bioactive compounds and their antioxidant capacity. It was found that the conditions for the maximum polyphenols extraction from grape pomaces and canes would be 50% ethanol, 80 °C for 30 min, for all samples rich in phenolic antioxidant active principles. The results also revealed that there are differences between our studied extracts (WP, RP, C), the white grape pomace extract revealing a higher antioxidant power in the DPPH and FRAP assays. These findings offer new perspectives for the use of winemaking by-products in the pharmaceutical and cosmetic field, and also can contribute to the reduction of environmental pollution.

## Figures and Tables

**Table 1:** Independent and dependent variables of the experimental design.

Independent Variables	Variation Level		
	−1	0	1
X1: Plant material	Red pomace (RP)	White pomace (WP)	Canes (C)
X2: Ethanol concentration	40	50	60
X3: Temperature	50	65	80
Dependent variable			
Y1: Total polyphenolic content (TPC)			

**Table 2:** Experimental design matrix and results obtained for total polyphenols content (TPC).

Experiment Name	Plant Material (X_1_)	Ethanol Ratio (%) (X_2_)	Temperature (°C) (X_3_)	TPC (Y)
N1	RP	40	50	17.12 ± 0.34
N2	RP	60	50	16.05 ± 0.32
N3	RP	40	80	25.17 ± 0.49
N4	RP	60	80	24.25 ± 0.46
N5	RP	50	65	24.27 ± 0.47
N6	WP	40	50	23.55 ± 0.46
N7	WP	60	50	22.15 ± 0.43
N8	WP	40	80	30.50 ± 0.59
N9	WP	60	80	31.35 ± 0.61
N10	WP	50	65	23.72 ± 0.41
N11	C	40	50	11.82 ± 0.24
N12	C	60	50	10.07 ± 0.21
N13	C	40	80	14.75 ± 0.29
N14	C	60	80	14.45 ± 0.29
N15	C	40	65	10.30 ± 0.21
N16	C	60	65	10.22 ± 0.21
N17	C	50	50	14.55 ± 0.29
N18	C	50	80	17.37 ± 0.34
N19	C	50	65	12.60 ± 0.25
N20	C	50	65	13.05 ± 0.26
N21	C	50	65	13.32 ± 0.27

Notes: TPC—total polyphenolic content, expressed as mg of gallic acid equivalents (GAE)/g dry plant material.

**Table 3:** Total polyphenol content of studied extracts.

Samples	TPC (mg GAE/g)	TFC (mg RE/g)	Caffeic acid Derivatives (mg CAE/g)	Tannins (mg GAE/g)
RP	32.00 ± 0.76	0.54 ± 0.07	9.11 ± 1.38	18.26 ± 1.73
WP	37.80 ± 0.19	1.89 ± 0.10	17.64 ± 1.35	12.56 ± 1.43
C	18.45 ± 0.48	0.33 ± 0.05	1.86 ± 0.08	13.14 ± 0.35

Each value is the mean ± SD of three independent measurements. TPC: Total polyphenols content; TFC: total flavonoid content; GAE: gallic acid equivalents; RE: rutin equivalents; CAE: caffeic acid equivalents.

**Table 4:** The polyphenolic content in the studied extracts (µg/g dry vegetal material).

Polyphenolic Compounds	*m*/*z* Value	tR ± SD (min)	WP (µg/g)	RP (µg/g)	C (µg/g)
Gallic acid	169	1.50 ± 0.01	128.19 ± 0.93	146.87 ± 1.13	39.57 ± 0.30
Protocatechuic acid	153	2.80 ± 0.01	57.17 ± 0.24	34.88 ± 0.12	11.69 ± 0.28
Caftaric acid	311	3.54 ± 0.05	<0.02	<0.02	<0.02
Gentisic acid	179	3.52 ± 0.04	<0.02	<0.02	-
Catechin	289	6.00 ± 0.03	539.14 ± 1.86	561.93 ± 4.07	413.40 ± 3.65
Vanillic acid	167	6.70 ± 0.01	-	45.00 ± 0.88	-
Syringic acid	197	8.40 ± 0.01	4.38 ± 0.12	72.22 ± 0.62	1.69 ± 0.01
Epicatechin	289	9.00 ± 0.01	513.52 ± 2.48	425.78 ± 4.22	278.90 ± 2.33
*p*-Coumaric acid	163	9.48 ± 0.08	-	<0.02	-
Hyperoside	463	18.60 ± 0.12	<0.02	-	<0.02
Isoquercitrin	463	19.60 ± 0.10	29.70 ± 0.30	6.59 ± 0.09	3.50 ± 0.05
Rutin	609	20.20 ± 0.15	2.63 ± 0.07	-	2.63 ± 0.04
Quercitrin	447	23.64 ± 0.13	26.08 ± 0.37	-	-
Quercetin	301	26.80 ± 0.15	6.69 ± 0.02	9.99 ± 0.01	-
Luteolin	285	29.10 ± 0.19	3.50 ± 0.04	-	-

Each value is the mean ± SD of three independent measurements. WP—white pomace, RP—red pomace, C- canes. Values are the mean ± SD (n = 3). Note: “-“ means not found, below detection limit.

**Table 5:** Antioxidant activity of studied extracts.

Samples	DPPH		FRAP (µmolTE/g)	NHA mg CATE/g
	I% (131.25 μg/mL)	IC_50_ μg/mL (37.5–262.50 μg/mL)		
RP	57.96	112.02 ± 3.98	2652.50 ± 6.49	16.87 ± 2.71
WP	80.93	76.33 ± 2.67	3908.42 ± 12.85	13.06 ± 4.40
C	30.86	198.27 ± 4.31	997.81 ± 2.18	8.27 ± 0.80
Trolox	-	11.27 ± 0.04	-	

Each value is the mean ± SD of three independent measurements. TE: Trolox equivalents. CATE: Catechin equivalents.
